# Predicting positron emission tomography brain amyloid positivity using wearable sensor data and lifestyle factors

**DOI:** 10.1002/alz70856_100717

**Published:** 2025-12-25

**Authors:** Ken Aoshima, Kotaro Sasaki, Noriyuki Kimura, Teruaki Masuda

**Affiliations:** ^1^ Eisai Co., Ltd., Tokyo, Japan; ^2^ University of Tsukuba, Tsukuba, Ibaraki, Japan; ^3^ Oita University, Yufu, Oita, Japan

## Abstract

**Background:**

Developing a screening method for identifying individuals at higher risk of elevated brain amyloid burden is important to reduce costs and burden to patients in clinical trials on Alzheimer's disease or the clinical setting.

**Method:**

We developed machine learning models using prospective cohort study (USUKI cohort) data with objectively measured lifestyle factors for individuals with mild cognitive impairment(MCI) or subjective memory complaints to predict elevated brain amyloid burden on positron emission tomography. In total, 118 presented with MCI who underwent cognitive test and PiB‐PET at baseline were recruited in the current study. Three machine learning models (kernel support vector machine, Elastic Net, and logistic regression) were developed.

**Result:**

In Elastic Net, the mean area under the receiver operating characteristic curve of the model using objectively measured lifestyle factors alone was 0.70, whereas that of the models using wearable sensors in combination with demographic characteristics and health and life environment questionnaires was 0.79. Moreover, 22 variables were common to all machine learning models.

**Conclusion:**

Our machine learning models are useful for predicting elevated brain amyloid burden for MCI patients using readily‐available and noninvasive variables without the need to visit a hospital.

